# Bis(3-hydroxy­pyridine-κ*N*)bis­(3-nitro­benzoato-κ*O*)zinc(II)

**DOI:** 10.1107/S1600536809027147

**Published:** 2009-07-15

**Authors:** Jun-Hua Li, Jing-Jing Nie, Duan-Jun Xu

**Affiliations:** a Department of Chemistry, Zhejiang University, People’s Republic of China

## Abstract

The title complex, [Zn(C_7_H_4_NO_4_)_2_(C_5_H_5_NO)_2_], has site symmetry 2. The Zn^II^ ion is located on a crystallographic twofold rotation axis and assumes a distorted tetra­hedral ZnN_2_O_2_ coordination geometry. Mol­ecules are linked by an inter­molecular O—H⋯O hydrogen bond and π–π stacking inter­actions between pyridine rings [centroid–centroid speparation 3.594 (1) Å].

## Related literature

For general background, see: Su & Xu (2004 ▶); Xu *et al.* (2007 ▶). For a related structure, see: Yan *et al.* (2008 ▶).
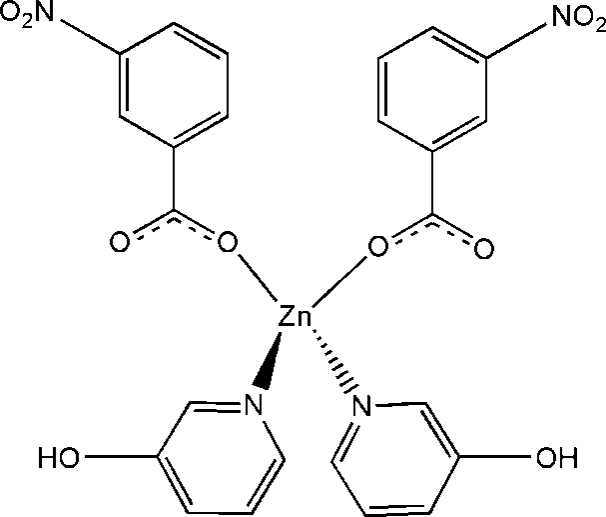


## Experimental

### 

#### Crystal data

[Zn(C_7_H_4_NO_4_)_2_(C_5_H_5_NO)_2_]
*M* *_r_* = 587.79
Monoclinic, 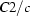

*a* = 22.992 (4) Å
*b* = 7.2412 (12) Å
*c* = 15.797 (3) Å
β = 111.584 (5)°
*V* = 2445.6 (8) Å^3^
*Z* = 4
Mo *K*α radiation
μ = 1.07 mm^−1^
*T* = 294 K
0.33 × 0.30 × 0.24 mm

#### Data collection

Rigaku R-AXIS RAPID IP diffractometer
Absorption correction: multi-scan (*ABSCOR*; Higashi, 1995 ▶) *T*_min_ = 0.655, *T*_max_ = 0.770
10172 measured reflections
2179 independent reflections
2038 reflections with *I* > 2σ(*I*)
*R*_int_ = 0.023

#### Refinement

*R*[*F* ^2^ > 2σ(*F* ^2^)] = 0.028
*wR*(*F* ^2^) = 0.080
*S* = 1.18
2179 reflections
177 parameters
H-atom parameters constrained
Δρ_max_ = 0.22 e Å^−3^
Δρ_min_ = −0.37 e Å^−3^

### 

Data collection: *PROCESS-AUTO* (Rigaku, 1998 ▶); cell refinement: *PROCESS-AUTO*; data reduction: *CrystalStructure* (Rigaku/MSC, 2002 ▶); program(s) used to solve structure: *SIR92* (Altomare *et al.*, 1993 ▶); program(s) used to refine structure: *SHELXL97* (Sheldrick, 2008 ▶); molecular graphics: *ORTEP-3 for Windows* (Farrugia, 1997 ▶); software used to prepare material for publication: *WinGX* (Farrugia, 1999 ▶).

## Supplementary Material

Crystal structure: contains datablocks I, global. DOI: 10.1107/S1600536809027147/bx2223sup1.cif

Structure factors: contains datablocks I. DOI: 10.1107/S1600536809027147/bx2223Isup2.hkl

Additional supplementary materials:  crystallographic information; 3D view; checkCIF report

## Figures and Tables

**Table 1:** Selected bond lengths (Å)

Zn—N1	2.0486 (16)
Zn—O2	1.9527 (13)

**Table 2:** Hydrogen-bond geometry (Å, °)

*D*—H⋯*A*	*D*—H	H⋯*A*	*D*⋯*A*	*D*—H⋯*A*
O1—H1*A*⋯O3^i^	0.91	1.73	2.642 (2)	174

Symmetry code: (i) 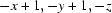.

**Fig. 1.**
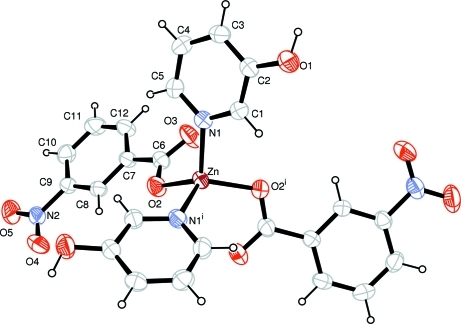
The molecular structure of the title compound with 40% probability displacement (arbitrary spheres for H atoms) [symmetry code: (i) 1 - x, y, -z + 1/2].

**Fig. 2.**
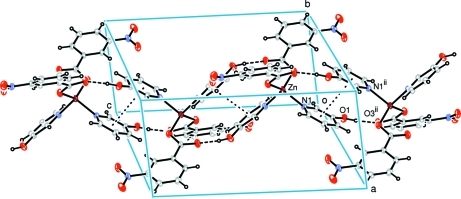
The unit cell packing diagram of the title compound showing π–π stacking between pyridine rings. Dashed and dotted lines indicate hydrogen bonding and π–π stacking, respectively [symmetry code: (ii) 1 - x, 1 - y, -z].

## supplementary crystallographic information

### Comment

As part of our ongoing investigation on the nature of π–π stacking (Su & Xu, 2004; Xu *et al.*, 2007), the title complex with pyridine ligand has recently been prepared in the laboratory, and its crystal structure is reported here.

The molecule has site symmetry 2, the Zn^II^ cation located on a twofold axis is coordinated by two hydroxypyridine ligands and two nitrobenzoate anions with a distorted tetrahedral geometry (Fig. 1 and Table 1). The O—Zn—O bond angle of 120.90 (9)° is much larger than the N—Zn—N bond angle of 101.72 (6)°. The partially overlapped arrangement of parallel pyridine rings is observed in the crystal structure (Fig. 2), the face-to-face separation of 3.594 (1) Å between N1-pyridine and N1^ii^-pyridine rings [symmetry code: (ii) 1 - *x*, 1 - *y*, -*z*] suggests the existence of π–π stacking between the parallel pyridine rings, similar to the situation found in *catena*-[(µ_2_-3,5-dinitro-2-oxybenzoato)(µ_2_-3-hydroxypyridine)- copper(II)] (Yan *et al.*, 2008). Intermolecular O—H···O hydrogen bond between hydroxyl and carboxyl groups is also present in the crystal structure (Table 2).

### Experimental

A water–ethanol solution (20 ml, 1:1) of 3-nitrobenzoic acid (0.17 g, 1 mmol), sodium carbonate (0.075 g, 0.7 mmol), 3-hydroxypyridine (0.19 g, 2 mmol) and zinc chloride (0.067 g, 0.5 mmol) was refluxed for 6 h. After cooling to room temperature the solution was filtered. The single crystals of the title compound were obtained from the filtrate after 4 d.

### Refinement

Hydroxy H atom was located in a difference Fourier map and was refined as riding in as-found relative position, *U*_iso_(H) = 1.5*U*_eq_(O). Aromatic H atoms were placed in calculated positions with C—H = 0.93 Å and were refined in riding mode with *U*_iso_(H) = 1.2*U*_eq_(C).

### Crystal data

**Table:**

[Zn(C_7_H_4_NO_4_)_2_(C_5_H_5_NO)_2_]	*F*(000) = 1200
*M**_r_* = 587.79	*D*_x_ = 1.596 Mg m^−^^3^
Monoclinic, *C*2/*c*	Mo *K*α radiation, λ = 0.71073 Å
Hall symbol: -C 2yc	Cell parameters from 2092 reflections
*a* = 22.992 (4) Å	θ = 2.0–25.0°
*b* = 7.2412 (12) Å	µ = 1.07 mm^−^^1^
*c* = 15.797 (3) Å	*T* = 294 K
β = 111.584 (5)°	Block, colourless
*V* = 2445.6 (8) Å^3^	0.33 × 0.30 × 0.24 mm
*Z* = 4	

### Data collection

**Table:**

Rigaku R-AXIS RAPID IP diffractometer	2179 independent reflections
Radiation source: fine-focus sealed tube	2038 reflections with *I* > 2σ(*I*)
graphite	*R*_int_ = 0.023
Detector resolution: 10.0 pixels mm^-1^	θ_max_ = 25.2°, θ_min_ = 1.9°
ω scans	*h* = −27→27
Absorption correction: multi-scan (*ABSCOR*; Higashi, 1995)	*k* = −7→8
*T*_min_ = 0.655, *T*_max_ = 0.770	*l* = −18→18
10172 measured reflections	

### Refinement

**Table:**

Refinement on *F*^2^	Primary atom site location: structure-invariant direct methods
Least-squares matrix: full	Secondary atom site location: difference Fourier map
*R*[*F*^2^ > 2σ(*F*^2^)] = 0.028	Hydrogen site location: inferred from neighbouring sites
*wR*(*F*^2^) = 0.080	H-atom parameters constrained
*S* = 1.18	*w* = 1/[σ^2^(*F*_o_^2^) + (0.0461*P*)^2^ + 1.01*P*] where *P* = (*F*_o_^2^ + 2*F*_c_^2^)/3
2179 reflections	(Δ/σ)_max_ < 0.001
177 parameters	Δρ_max_ = 0.22 e Å^−^^3^
0 restraints	Δρ_min_ = −0.37 e Å^−^^3^

### Special details

**Table:**

Geometry. All e.s.d.'s (except the e.s.d. in the dihedral angle between two l.s. planes) are estimated using the full covariance matrix. The cell e.s.d.'s are taken into account individually in the estimation of e.s.d.'s in distances, angles and torsion angles; correlations between e.s.d.'s in cell parameters are only used when they are defined by crystal symmetry. An approximate (isotropic) treatment of cell e.s.d.'s is used for estimating e.s.d.'s involving l.s. planes.
Refinement. Refinement of *F*^2^ against ALL reflections. The weighted *R*-factor *wR* and goodness of fit *S* are based on *F*^2^, conventional *R*-factors *R* are based on *F*, with *F* set to zero for negative *F*^2^. The threshold expression of *F*^2^ > σ(*F*^2^) is used only for calculating *R*-factors(gt) *etc*. and is not relevant to the choice of reflections for refinement. *R*-factors based on *F*^2^ are statistically about twice as large as those based on *F*, and *R*- factors based on ALL data will be even larger.

### Fractional atomic coordinates and isotropic or equivalent isotropic displacement parameters (Å^2^)

**Table:**

	*x*	*y*	*z*	*U*_iso_*/*U*_eq_	
Zn	0.5000	0.60825 (4)	0.2500	0.03308 (13)	
N1	0.51519 (7)	0.4295 (2)	0.15973 (11)	0.0353 (4)	
N2	0.76573 (8)	0.9962 (2)	0.57621 (11)	0.0428 (4)	
O1	0.41195 (6)	0.2005 (2)	−0.04113 (10)	0.0502 (4)	
H1A	0.4151	0.1741	−0.0959	0.075*	
O2	0.57415 (6)	0.7413 (2)	0.32834 (9)	0.0430 (3)	
O3	0.57500 (8)	0.8529 (3)	0.19867 (10)	0.0582 (4)	
O4	0.73267 (8)	0.9305 (3)	0.61402 (11)	0.0597 (4)	
O5	0.81810 (8)	1.0587 (3)	0.61763 (12)	0.0639 (5)	
C1	0.46440 (9)	0.3618 (3)	0.09435 (13)	0.0376 (4)	
H1	0.4256	0.3830	0.0983	0.045*	
C2	0.46675 (9)	0.2612 (3)	0.02073 (12)	0.0360 (4)	
C3	0.52475 (9)	0.2297 (3)	0.01547 (13)	0.0386 (4)	
H3	0.5283	0.1659	−0.0335	0.046*	
C4	0.57718 (9)	0.2956 (3)	0.08477 (14)	0.0442 (5)	
H4	0.6166	0.2729	0.0834	0.053*	
C5	0.57162 (9)	0.3943 (3)	0.15562 (14)	0.0402 (5)	
H5	0.6075	0.4377	0.2016	0.048*	
C6	0.59870 (9)	0.8367 (3)	0.28242 (13)	0.0376 (4)	
C7	0.66049 (9)	0.9261 (3)	0.33534 (13)	0.0341 (4)	
C8	0.68297 (9)	0.9269 (3)	0.43004 (13)	0.0341 (4)	
H8	0.6590	0.8799	0.4613	0.041*	
C9	0.74167 (9)	0.9988 (3)	0.47648 (12)	0.0355 (4)	
C10	0.77887 (9)	1.0701 (3)	0.43250 (15)	0.0434 (5)	
H10	0.8185	1.1168	0.4654	0.052*	
C11	0.75552 (11)	1.0701 (3)	0.33837 (16)	0.0491 (5)	
H11	0.7795	1.1182	0.3073	0.059*	
C12	0.69704 (10)	0.9994 (3)	0.29032 (14)	0.0426 (5)	
H12	0.6818	1.0006	0.2270	0.051*	

### Atomic displacement parameters (Å^2^)

**Table:**

	*U*^11^	*U*^22^	*U*^33^	*U*^12^	*U*^13^	*U*^23^
Zn	0.03108 (18)	0.0418 (2)	0.02401 (18)	0.000	0.00732 (12)	0.000
N1	0.0354 (8)	0.0405 (9)	0.0294 (8)	0.0019 (7)	0.0112 (7)	−0.0008 (6)
N2	0.0417 (9)	0.0484 (11)	0.0331 (9)	0.0088 (8)	0.0074 (8)	−0.0057 (7)
O1	0.0417 (8)	0.0636 (10)	0.0403 (8)	−0.0044 (7)	0.0091 (6)	−0.0163 (7)
O2	0.0361 (7)	0.0557 (9)	0.0345 (7)	−0.0105 (6)	0.0097 (6)	−0.0003 (6)
O3	0.0593 (10)	0.0789 (11)	0.0279 (8)	−0.0163 (8)	0.0061 (7)	−0.0030 (7)
O4	0.0613 (10)	0.0864 (13)	0.0321 (8)	0.0003 (9)	0.0181 (8)	−0.0025 (8)
O5	0.0444 (9)	0.0838 (12)	0.0466 (9)	−0.0016 (8)	−0.0031 (7)	−0.0135 (9)
C1	0.0326 (9)	0.0459 (11)	0.0346 (10)	0.0012 (8)	0.0128 (8)	−0.0037 (8)
C2	0.0387 (10)	0.0363 (10)	0.0311 (9)	−0.0001 (8)	0.0107 (8)	−0.0001 (8)
C3	0.0471 (11)	0.0372 (10)	0.0358 (10)	0.0031 (8)	0.0201 (9)	−0.0017 (8)
C4	0.0369 (10)	0.0519 (13)	0.0477 (12)	0.0064 (9)	0.0203 (9)	−0.0001 (10)
C5	0.0323 (9)	0.0470 (12)	0.0376 (11)	0.0017 (8)	0.0086 (8)	−0.0014 (8)
C6	0.0374 (10)	0.0423 (11)	0.0313 (10)	−0.0018 (8)	0.0106 (8)	−0.0048 (8)
C7	0.0366 (10)	0.0374 (10)	0.0292 (9)	−0.0012 (8)	0.0131 (8)	−0.0014 (7)
C8	0.0339 (9)	0.0399 (10)	0.0310 (10)	−0.0013 (8)	0.0148 (8)	−0.0011 (7)
C9	0.0359 (9)	0.0382 (10)	0.0310 (10)	0.0033 (8)	0.0106 (8)	−0.0036 (8)
C10	0.0329 (10)	0.0482 (12)	0.0478 (12)	−0.0067 (9)	0.0133 (9)	−0.0054 (9)
C11	0.0477 (12)	0.0589 (14)	0.0498 (13)	−0.0082 (10)	0.0287 (10)	0.0030 (10)
C12	0.0491 (11)	0.0501 (12)	0.0322 (10)	−0.0043 (9)	0.0193 (9)	0.0011 (9)

### Geometric parameters (Å, °)

**Table:**

Zn—N1	2.0486 (16)	C3—C4	1.381 (3)
Zn—N1^i^	2.0486 (16)	C3—H3	0.9300
Zn—O2	1.9527 (13)	C4—C5	1.373 (3)
Zn—O2^i^	1.9527 (13)	C4—H4	0.9300
N1—C1	1.335 (2)	C5—H5	0.9300
N1—C5	1.347 (3)	C6—C7	1.504 (3)
N2—O4	1.223 (2)	C7—C12	1.390 (3)
N2—O5	1.226 (2)	C7—C8	1.392 (3)
N2—C9	1.465 (3)	C8—C9	1.379 (3)
O1—C2	1.353 (2)	C8—H8	0.9300
O1—H1A	0.9147	C9—C10	1.385 (3)
O2—C6	1.274 (2)	C10—C11	1.383 (3)
O3—C6	1.237 (2)	C10—H10	0.9300
C1—C2	1.390 (3)	C11—C12	1.377 (3)
C1—H1	0.9300	C11—H11	0.9300
C2—C3	1.385 (3)	C12—H12	0.9300
			
O2—Zn—O2^i^	120.90 (9)	C3—C4—H4	119.7
O2—Zn—N1	114.84 (6)	N1—C5—C4	121.20 (18)
O2^i^—Zn—N1	101.72 (6)	N1—C5—H5	119.4
O2—Zn—N1^i^	101.72 (6)	C4—C5—H5	119.4
O2^i^—Zn—N1^i^	114.84 (6)	O3—C6—O2	123.17 (18)
N1—Zn—N1^i^	101.64 (9)	O3—C6—C7	120.52 (18)
C1—N1—C5	118.48 (16)	O2—C6—C7	116.28 (16)
C1—N1—Zn	116.48 (12)	C12—C7—C8	119.53 (18)
C5—N1—Zn	124.53 (13)	C12—C7—C6	120.34 (17)
O4—N2—O5	123.22 (18)	C8—C7—C6	120.03 (17)
O4—N2—C9	118.26 (17)	C9—C8—C7	118.50 (17)
O5—N2—C9	118.52 (18)	C9—C8—H8	120.8
C2—O1—H1A	112.0	C7—C8—H8	120.8
C6—O2—Zn	111.90 (12)	C8—C9—C10	122.55 (18)
N1—C1—C2	123.20 (17)	C8—C9—N2	118.40 (17)
N1—C1—H1	118.4	C10—C9—N2	119.04 (17)
C2—C1—H1	118.4	C11—C10—C9	118.19 (19)
O1—C2—C3	124.38 (17)	C11—C10—H10	120.9
O1—C2—C1	117.54 (17)	C9—C10—H10	120.9
C3—C2—C1	118.08 (17)	C12—C11—C10	120.46 (19)
C4—C3—C2	118.33 (17)	C12—C11—H11	119.8
C4—C3—H3	120.8	C10—C11—H11	119.8
C2—C3—H3	120.8	C11—C12—C7	120.76 (19)
C5—C4—C3	120.66 (18)	C11—C12—H12	119.6
C5—C4—H4	119.7	C7—C12—H12	119.6
			
O2—Zn—N1—C1	−168.19 (13)	Zn—O2—C6—C7	−172.72 (13)
O2^i^—Zn—N1—C1	−35.82 (15)	O3—C6—C7—C12	−11.6 (3)
N1^i^—Zn—N1—C1	82.92 (14)	O2—C6—C7—C12	166.42 (19)
O2—Zn—N1—C5	3.54 (18)	O3—C6—C7—C8	171.9 (2)
O2^i^—Zn—N1—C5	135.91 (16)	O2—C6—C7—C8	−10.1 (3)
N1^i^—Zn—N1—C5	−105.36 (17)	C12—C7—C8—C9	−0.9 (3)
O2^i^—Zn—O2—C6	−63.65 (13)	C6—C7—C8—C9	175.64 (17)
N1—Zn—O2—C6	58.89 (15)	C7—C8—C9—C10	0.1 (3)
N1^i^—Zn—O2—C6	167.73 (14)	C7—C8—C9—N2	−178.86 (17)
C5—N1—C1—C2	−1.9 (3)	O4—N2—C9—C8	0.0 (3)
Zn—N1—C1—C2	170.31 (15)	O5—N2—C9—C8	179.42 (18)
N1—C1—C2—O1	−179.81 (18)	O4—N2—C9—C10	−179.00 (19)
N1—C1—C2—C3	0.2 (3)	O5—N2—C9—C10	0.4 (3)
O1—C2—C3—C4	−178.31 (19)	C8—C9—C10—C11	0.6 (3)
C1—C2—C3—C4	1.7 (3)	N2—C9—C10—C11	179.55 (19)
C2—C3—C4—C5	−1.9 (3)	C9—C10—C11—C12	−0.5 (3)
C1—N1—C5—C4	1.8 (3)	C10—C11—C12—C7	−0.3 (3)
Zn—N1—C5—C4	−169.79 (15)	C8—C7—C12—C11	1.0 (3)
C3—C4—C5—N1	0.1 (3)	C6—C7—C12—C11	−175.53 (19)
Zn—O2—C6—O3	5.2 (3)		

Symmetry codes: (i) −*x*+1, *y*, −*z*+1/2.

### Hydrogen-bond geometry (Å, °)

**Table:**

*D*—H···*A*	*D*—H	H···*A*	*D*···*A*	*D*—H···*A*
O1—H1A···O3^ii^	0.91	1.73	2.642 (2)	174

Symmetry codes: (ii) −*x*+1, −*y*+1, −*z*.
